# Validation of a simplified-geometry model of inhaled formoterol pharmacodynamics in asthmatic patients

**DOI:** 10.3389/fphys.2022.1018050

**Published:** 2022-12-02

**Authors:** Simona Panunzi, Claudio Gaz, Fabio Cibella, Andrea De Gaetano

**Affiliations:** ^1^ Laboratorio di Biomatematica (BioMatLab), Istituto di Analisi dei Sistemi ed Informatica “A. Ruberti”, Consiglio Nazionale delle Ricerche, Roma, Italy; ^2^ Faculty of Science, Engineering and Computing, Department of Mechanical Engineering, Kingston University, London, United Kingdom; ^3^ Istituto per la Ricerca e l’Innovazione Biomedica, Consiglio Nazionale delle Ricerche, Palermo, Italy; ^4^ Department of Biomatics, Óbuda University, Budapest, Hungary

**Keywords:** mathematical model, FEV1, clinical trial simulation, formoterol, bronchial asthma, model validation

## Abstract

PharmacoKinetics (PK) and PharmacoDynamics (PD) mathematical models of inhaled bronchodilators represent useful tools for understanding the mechanisms of drug action and for the individuation of therapy regimens. A PK/PD model for inhaled bronchoactive compounds was previously proposed, incorporating a simplified-geometry approach: the key feature of that model is a mixed compartmental and spatially distributed representation of the kinetics, with the direct computation of representative flow rates from Ohm’s law and bronchial diameter profiles. The aim of the present work is the enrichment and validation of this simplified geometry modeling approach against clinical efficacy data. The improved model is used to compute airflow response to treatment for each single virtual patient from a simulated population and it is found to produce very good fits to observed FEV_1_ profiles. The model provides a faithful quantitative description of the increasing degree of improvement with respect to basal conditions with continuing administration and with increasing drug dosages, as clinically expected.

## Introduction

Formoterol is a rapid-onset, long-acting β_2_ agonist, typically administered by inhalation in combination with low-to-high doses of corticosteroids for control-based asthma management in asthmatic patients [Global Strategy for Asthma Management and Prevention—GINA—2022 update (Ginasthma, 2022)]. Formoterol is also used as reliever medication in association with low dose corticosteroids. In fact, different studies have demonstrated that it is effective as short-acting β_2_ agonists providing rapid bronchodilation within 1–3 min of administration and that its duration of action extends to up to 12 h after inhalation (Hekking et al., 1990; Palmqvist et al., 1997; Ringdal et al., 1998; Seberova and Andersson, 2000; Welsh and Ctes, 2010). Moreover, results from previous studies (Castle et al., 1993; Nelson et al., 2006) provide support to its use in combination with inhaled corticosteroids (ICSs) and demonstrate its greater effectiveness in comparison with salmeterol. Other studies (Faulds et al., 1991; Politiek et al., 1999) compare the efficacy of formoterol against both short- and long-acting β_2_ agonists, concluding that formoterol and salbutamol have roughly the same efficacy.

The Aerolizer^®^, a dry powder inhaler (DPI), has been introduced for use for the administration of formoterol instead of the traditional metered-dose inhalers (MDI), which deliver a formoterol solution aerosol. DPIs are particularly useful for patients with difficult coordination in inspiration and assure the correct pharmacological dose administration in these patients.

Several studies prove the efficacy and safety of formoterol delivered *via* Aerolizer (Bensch et al., 2001; Pleskow et al., 2003), when compared with albuterol *via* Metered Dose Inhaler. Moreover, formoterol DPI provides an equivalent bronchodilating effect with respect to formoterol MDI in asthmatic patients (Bousquet et al., 2005).

In a previous work Gaz et al. (2012), a mathematical approach to the description of the fate of a compound administered by the inhalation of an aerosolized cloud of droplets was presented. In that (pharmacokinetic/pharmacodynamic—PK/PD) model the relevant pharmacokinetics was represented with the use of five compartments. Two of them were aggregated compartments representing the bronchial tree and associated muscle divided in turn into sub-compartments representing the spatial dimension along the depth of the bronchial tree. The model proposed in that work differs from traditional PK/PD models by introducing a simplified geometrical and functional description of the bronchial tree, leading to the direct computation of the approximate airflow. Anatomical and geometrical features, such as bronchial branching and smooth muscle distribution, are taken into account.

This approach takes a middle road between pure compartmental modeling of the respiratory system, along with the blood-to-tissue distribution of substances (giving rise to a system of nonlinear ordinary differential equations, ODE’s), and full Computational Fluid Dynamics (CFD) (Donovan et al., 2012; Vos et al., 2013; De Backer et al., 2015), in which the respiratory system of a given subject is geometrically modeled in three-dimensional space, typically on the basis of CT scans or other medical images.

In this simplified geometry approach (Gaz et al., 2012), modeling of the bronchial region takes into account a partial differential process in one spatial variable, because many crucial PK and PD features depend on the depth into the bronchial tree. The distribution of anatomical and physiological characteristics down the bronchial tree can thus be taken into account in order to obtain a physiologically-based representation of the pharmacodynamics effect of inhaled bronchodilators.

The simulations obtained with the simplified geometry model agreed very well with expected behavior of the time-course of forced expiratory volume in the first second (FEV_1_) after the administration of inhaled medication. The main advantage of the new model with respect to standard PK/PD formulations was thought to be in the closer mechanistic approximation to the actual physiology of respiration and to the corresponding drug particle deposition, whereas its main advantage with respect to full-blown Computational Fluid Dynamics was the possibility of representing many subjects within the limits of a reasonable computational burden.

The aim of the present study is therefore that of validating the simplified geometry modeling approach against clinical efficacy data. In order to do so, we will proceed to build a Clinical Trial Simulation. The reasoning proceeds in three steps: a simulated population reflecting the demographics and the disease–related characteristics of Pleskow’s study population (Pleskow et al., 2003) is created (*The Simulation step*); a modified version of the simplified geometry PK/PD model (Gaz et al., 2012) is used to compute airflow response to treatment for each single virtual patient (*The Modelling step*); the FEV_1_ results obtained in virtual samples are compared with those obtained in the real sample of adolescent and adult asthmatics studied by Pleskow et al. (2003) in order to validate the model in its new formulation (*The Parameter Estimation step*).

## Methods

### Pleskow’s study design

Pleskow’s study (Pleskow et al., 2003) was a multicenter, randomized, double-blind, double dummy, parallel-group study. The aim of the study was that of comparing efficacy and tolerability of twice-daily formoterol dry powder 12 μg and 24 μg (Foradil) delivered *via* Aerolizer inhaler against four times daily albuterol (salbutamol) 180 μg delivered *via* metered dose inhaler (MDI). A matching placebo group was also used for comparison. Adolescent and adult patients with mild-to-moderate asthma were screened and followed for a run-in period before being randomized to one of the above four treatment groups. The double-blind treatment period lasted 12-week. The design of the study contemplated a spirometry evaluation, consisting of FEV_1_, Forced Vital Capacity (FVC) and maximum mid-expiratory flow (FEF_25–75%_) evaluated at 0, 5, 15, and 30 min and hourly from 1 to 12 h—post-dose—at weeks 0, 4, 8, 12 and at the final visit. The primary efficacy end-point of the study was the serial FEV_1_ values over the 12-week study (from week 0 to week 12). The measurements of FEV_1_ over the 12 h, at week 0 and week 12, are related to the post treatment administration period (post-dose).

Results from the study showed that FEV_1_ measurements from the formoterol treatment groups were clinically and statistically higher than those from the placebo group. For more details of this study refer to Pleskow et al. (2003).

### The Simulation step: Reproducing the target population characteristics

The first objective of the present work was that of virtually reproducing Pleskow’s populations in terms of both demographic and disease-associated characteristics. In the present work only the groups undergoing treatment with formoterol (12 μg or 24 μg) were considered. The demographic covariates taken into account were age, gender and height; the covariates associated with the disease severity were expected FEV_1_, as function of the above demographic characteristics according to the Quanjer GLI-2012 regression equations (Quanjer et al., 2012), as well as fraction of predicted FEV_1_. Reference mean and standard deviation values for the above variables measured at baseline, were taken from Table 1 of Pleskow et al. (2003). 10,000 virtual patients were generated so as to obtain age, FEV_1_, percentages of predicted FEV_1_ and gender distributions as close as possible (in terms of averages and standard deviations) to those observed in Pleskow’s patient sample. The covariate distributions were simulated under parametric assumptions as follows. For age, a truncated normal distribution was adopted. A bi-modal normal distribution was used to generate the percentage of predicted FEV_1_ with most of the subjects near the 40% or the 80% limits and few subjects in between: the presence of two subpopulations was therefore hypothesized, one presenting with a low degree of basal obstruction and one presenting with a more severe air obstruction (this was necessary in order to match the observed sample characteristics). Even if such a distribution could in principle appear to be little plausible, however there is no unimodal probability distribution able to reproduce the observed Pleskow’s data: patients within a narrow range of percentage of predicted FEV_1_ (from 40% to 80%), with an average value of 66.5 and a quite large standard deviation of 16.3%. Males and females were generated in the same proportions as reported by Pleskow, while the distribution of FEV_1_ was generated from the distributions of the expected FEV_1_ (*ExpectedFEV*_*1*_) and of the percentage of predicted FEV_1_ (*PercFEV*_*1*_) according to the formula:

$$FEV_{1}=\frac{ExpectedFEV_{1}\times PercFEV_{1}}{100}$$

The above computation refers to the baseline condition, that is before the administration of formoterol.

Because Table 1 in Pleskow et al. (2003) did not report population averages and standard deviations of heights in females and males (height being a necessary predictor for the computation of the expected FEV_1_), these values, for the two populations, were found by minimizing, with respect to θ, the following loss function:

$$L\left(\theta \right)={\left(\frac{TargetMeanFEV_{1}-MeanFEV_{1}\left(\theta \right)}{MeanFEV_{1}\left(\theta \right)}\right)}^{2}+{\left(\frac{{TargetStDevFEV}_{1}-StDevFEV_{1}\left(\theta \right)}{StDevFEV_{1}\left(\theta \right)}\right)}^{2}$$

where θ is the parameter vector containing the generating population height and standard deviation parameters for males and females. These parameters were used to sample the heights from two normal distributions, one for females and one for males. Once the gender was generated according to the Pleskow’s gender distribution, height was sampled with one or the other distribution according to the generated gender. In the above loss function *TargetMeanFEV*_*1*_ and *TargetStDevFEV*_*1*_ are the mean and standard deviation, respectively, of the observed mean and standard deviation reported for the Pleskow’s sample. *MeanFEV*_*1*_(θ) and *StDevFEV*_*1*_(θ) are functions of θ by means of the variable *ExpectedFEV*_*1*_ which depends indeed on the height. The optimization procedure was started with different values of mean and standard deviation for males and females: 170 ± 27 and 160 ± 22 cm, respectively.

Notice that truncated distributions were used because the originally enrolled patient samples were confined to given age and fractional expected FEV_1_ brackets.

Table 1 reports the average values and standard deviations (or percentages as appropriate) of the observed (from Pleskow’s patient sample) and simulated (virtual subjects from the simulation step) demographic and disease-related variables. Table 2 reports the average observed FEV_1_ at post-dose times both at week 0 and week 12.

**TABLE 1:** Baseline Pleskow’s population characteristics along with the obtained average characteristics from the two simulated populations (10,000 patients each).

A	Pleskow population formoterol 12 μg	Simulated population formoterol 12 μg
No. of patients	139	10,000
Gender Male (%)	69 (50%)	4946 (49.5%)
Female (%)	70 (50%)	5054 (50.5%)
Age [yr] (SD)	32.6 (13.9)	32.5 (14.0)
FEV_1_ [L]	2.3 (0.8)	2.3 (0.8)
% of predicted FEV_1_	66.5 (16.3)	66.5 (16.3)

**B**	Pleskow population Formoterol 24 μg	Simulated population Formoterol 24 μg
No. of patients	136	10,000
Gender Male (%)	76 (56%)	5585 (55.9%)
Female (%)	60 (44%)	4415 (44.1%)
Age [yr] (SD)	32.6 (14.9)	32.6 (13.9)
FEV_1_ [L]	2.4 (0.8)	2.4 (0.8)
% of predicted FEV_1_	65.1 (15.6)	65.1 (15.5)

**TABLE 2:** Pleskow’s post-dose observed FEV_1_ on the first day (week 0) and at week 12 of double-blind treatment with formoterol 12 μg or 24 μg delivered *via* Aerolizer.

Treatment 1: 12 μg formoterol				Treatment 2: 24 μg formoterol			
Week 0		Week 12		Week 0		Week 12	
Time post-dose [hr]	FEV1 [L]	Time post-dose [hr]	FEV1 [L]	Time post-dose [hr]	FEV1 [L]	Time post-dose [hr]	FEV_1_ [L]
0	2.31	0	2.59	0	2.37	0	2.68
0.083333	2.71	0.083333	2.78	0.083333	2.86	0.083333	2.92
0.25	2.79	0.25	2.87	0.25	2.99	0.25	3
0.5	2.86	0.5	2.91	0.5	3.06	0.5	3.07
1	2.92	1	2.97	1	3.14	1	3.14
2	2.97	2	2.97	2	3.19	2	3.14
3	3.01	3	3	3	3.22	3	3.16
4	3	4	2.96	4	3.2	4	3.13
5	2.99	5	2.92	5	3.19	5	3.08
6	2.96	6	2.86	6	3.15	6	3.03
7	2.91	7	2.83	7	3.13	7	3.02
8	2.92	8	2.8	8	3.11	8	3.02
9	2.89	9	2.83	9	3.11	9	2.97
10	2.86	10	2.8	10	3.09	10	2.93
11	2.86	11	2.8	11	3.07	11	2.93
12	2.87	12	2.79	12	3.05	12	2.92

### The Modelling step: A mixed compartmental and distributed PK/PD model

#### The pharmacokinetics equations

In Gaz et al. (2012) a mixed compartmental and distributed PK/PD model was proposed, where the pharmacodynamic effect was derived from the (distributed) concentrations of drug in the effect compartment (bronchial muscle). The model equations are reported below:

$$\frac{dP\left(t\right)}{dt}=-\left(k_{xp}+k_{mp}\right)P\left(t\right)-k_{gp}P\left(t\right)+\frac{k_{pg}G\left(t\right)+k_{pm}\int_{z_{0}}^{z_{\max}}M\left(z,t\right)dz}{V_{distr}W},P\left(0\right)=0$$ (1)

$$\frac{dG\left(t\right)}{dt}=-k_{pg}G\left(t\right)-k_{xg}G\left(t\right)+k_{gp}P\left(t\right)V_{distr}W,G\left(0\right)=\eta \left(1-\rho \right)b_{g}D_{0}$$ (2)

$$\frac{dB\left(z,t\right)}{dt}=-k_{mb}B\left(z,t\right)+k_{bm}M\left(z,t\right)+\varphi_{w}\frac{\partial^{2}B\left(z,t\right)}{\partial^{2}z^{2}},\;B\left(z,0\right)=f\left(z\right)\eta \rho b_{b}D_{0}$$ (3)

$$\frac{dM\left(z,t\right)}{dt}=-\left(k_{pm}+k_{bm}\right)M\left(z,t\right)+k_{mb}B\left(z,t\right)+\frac{k_{mp}}{\int dz}P\left(t\right)\cdot V_{distr}W,\;M\left(z,0\right)=0\forall z\in \left[z_{\min},z_{\max}\right]$$ (4)

$$\frac{dU\left(t\right)}{dt}=\psi \;k_{xp}P\left(t\right)\;V_{distr}W,\;U\left(0\right)=0$$ (5)

The above model formulation hypothesizes that the sprayed dose *D*_0_ is split into two parts, one being the amount actually delivered to the mouth *ηD*_0_, and the other one (1-η)*D*_0_ being the fraction of the active compound dose *D*_0_ remaining in the device itself. A fraction *ρb*_*b*_ (where *b*_*b*_ is the drug’s bronchial bioavailability) of the delivered dose reaches the spatially distributed compartment *B* (Eq. 3), spreads instantaneously over the entire bronchial tree according to a probability density *f*(*z*), and is transferred to the bronchial muscle fibres (spatially distributed compartment *M*, Eq. 4) with apparent first order transfer rate *k*_*mb*_. The remaining fraction (*1-ρ*)*η b*_*g*_ *D*_0_ of the delivered drug is transferred to the bioavailable gastrointestinal drug depot *G* (Eq. 2), where *b*_*g*_ is the gastrointestinal drug bioavailability.

The probability density *f*(*z*), appearing in the boundary condition of Eq. 3, depends on the physical characteristics (aero and hydrodynamics) of the aerosolized particles. The symbol *z* represents the single spatial dimension, expressed as a standardized distance of a point along the bronchial tree from the end of the larynx. The probability density of deposition *f* depends therefore on *z*. After its deposition, the compound diffuses in time along the bronchial tree with a diffusion coefficient *φ*_*w*_ and is locally absorbed from the mucosa into the bronchial muscle compartment.

The compound is then transferred from compartments *G* and *M* into the plasma compartment *P* (Eq. 1) with apparent first order transfer rates *k*_*pg*_ and *k*_*pm*_. From plasma, the drug is eliminated at a rate *k*_*xp*_ into the compartment *U* (Eq. 5), representing the quantity of formoterol in the urine, with *ψ* indicating the recovery fraction of the drug. The parameter *k*_*gp*_ represents hepato-biliary extraction whereas the parameter *k*_*xg*_ represents partial gastrointestinal elimination: in the present simulation both are set to zero. Moreover, given the small amount of drug actually transferred to bronchial smooth muscle and the relatively good blood perfusion of the muscle itself, the parameter *k*_*bm*_ was also set to zero. Finally, although the model allows bidirectional compound exchange between the P and M compartments, so that available drug could in principle be transferred from plasma to bronchial muscle, also the parameter *k*_*mp*_ was set to 0, reflecting the very minor role of back-transfer of active substance from plasma to the effect site.

#### The pharmacodynamics equations

All the hypotheses on which the mathematical representation of the geometrical and behavioural features of the bronchial system is based upon are detailed in Gaz et al. (2012). The key assumptions are that bronchioles at the same relative distance down the bronchial tree present the same structural features and behaviour and that the drug effect on a subject depends on the position, along the bronchial tree, where the compound is deposited (Gaz et al., 2012). Briefly, at the bronchial level, β_2_-adrenergic receptors, when stimulated by the presence of the drug, determine local bronchodilatation: the diameters of the bronchioles increase with a resulting decrease of airway resistances (to which airflow is inversely related by the first Ohm’s law). The model describes the direct computation of the approximate Forced Expiratory Volume in 1 s (FEV_1_), as the volume moved in 1 s under constant expiratory pressure, assuming constant bronchial geometry and elastic recoil, given the PK of the substance.

We report below the main equations and modelling assumptions.

Let *δ*_*m*_(*z*,0) be the bronchial profile at time 0 representing the degree of patency of the airways, expressed as fraction of normal bronchial diameter at each *z*. For a healthy subject, the disease profile is identically equal to 1; in case of a broncho-constricted subject, as for example in an asthmatic subject, the constriction profile has been represented by

$$\delta_{m}\left(z,0\right)=1-Ae^{-\frac{{\left(z-b\right)}^{2}}{2c^{2}}}$$ (6)

where *A* is the maximal restriction amplitude (as a fraction of 1), *b* is the position of the maximal bronchial restriction along *z* and *c* is the standard deviation of the Gaussian curve which determines the width of the constriction. In the original work (Gaz et al., 2012), the drug dynamic effect over time was associated with the bronchial muscle content of the active principle as follows:

$$\frac{d\delta_{m}\left(z,t\right)}{dt}=k_{mor}\left(\delta_{m}\left(z,0\right)-\delta_{m}\left(z,t\right)\right)+k_{med}h\left(z\right)M\left(z,t\right)\left(1-\delta_{m}\left(z,t\right)\right)$$ (7)

where the initial condition in the above equation is the disease profile described in Eq. 6; *M*(*z*,*t*) is the drug content density of bronchial muscle at time *t* and position *z*, as computed from the kinetic part of the model (Eq. 4); *k*_*mor*_ is a constant representing the degree of the subject’s morbidity (as the rate of spontaneous return of the bronchial diameter towards its diseased profile), while *k*_*med*_ represents the medicinal efficacy of the drug (as the rate of modification of the bronchial profile towards unity, or towards 100% patency).

In the present formulation, instead, in order to reproduce the observed initial fast rise and subsequent progressive further rise followed by gradual fall of the observed effect, a modification of Eq. 7 proved necessary: while in Eq. 7 the compound passes directly from the bronchial mucosa into the muscle compartment effect site, here it is hypothesized that two parallel delay compartments (one faster and one slower) have to be interposed between the mucosa and the muscle compartment:

$$\frac{d\delta_{m}\left(z,t\right)}{dt}=k_{mor}\exp\left(-\lambda_{1}E\left(z,t\right)\right)\left(\delta_{m}\left(z,0\right)-\delta_{m}\left(z,t\right)\right)+k_{med}\frac{h\left(z\right)}{h_{\max}}(1-\exp\left(-\lambda_{2}E\left(z,t\right)\right)\left(1-\delta_{m}\left(z,t\right)\right)$$ (8)

$$\frac{dE_{1}\left(z,t\right)}{dt}=k_{1m}M\left(z,t\right)-k_{e1}E_{1}\left(z,t\right),E_{1}\left(z,0\right)=0\forall z\in \left[z_{\min},z_{\max}\right]$$ (9)

$$\frac{dE_{2}\left(z,t\right)}{dt}=k_{2m}M\left(z,t\right)-k_{e2}E_{2}\left(z,t\right),E_{2}\left(z,0\right)=0\forall z\in \left[z_{\min},z_{\max}\right]$$ (10)

$$\frac{dE\left(z,t\right)}{dt}=k_{e1}E_{1}\left(z,t\right)+k_{e2}E_{2}\left(z,t\right)-k_{xe}E\left(z,t\right),E\left(z,0\right)=0\forall z\in \left[z_{\min},z_{\max}\right]$$ (11)

**FIGURE 1:**
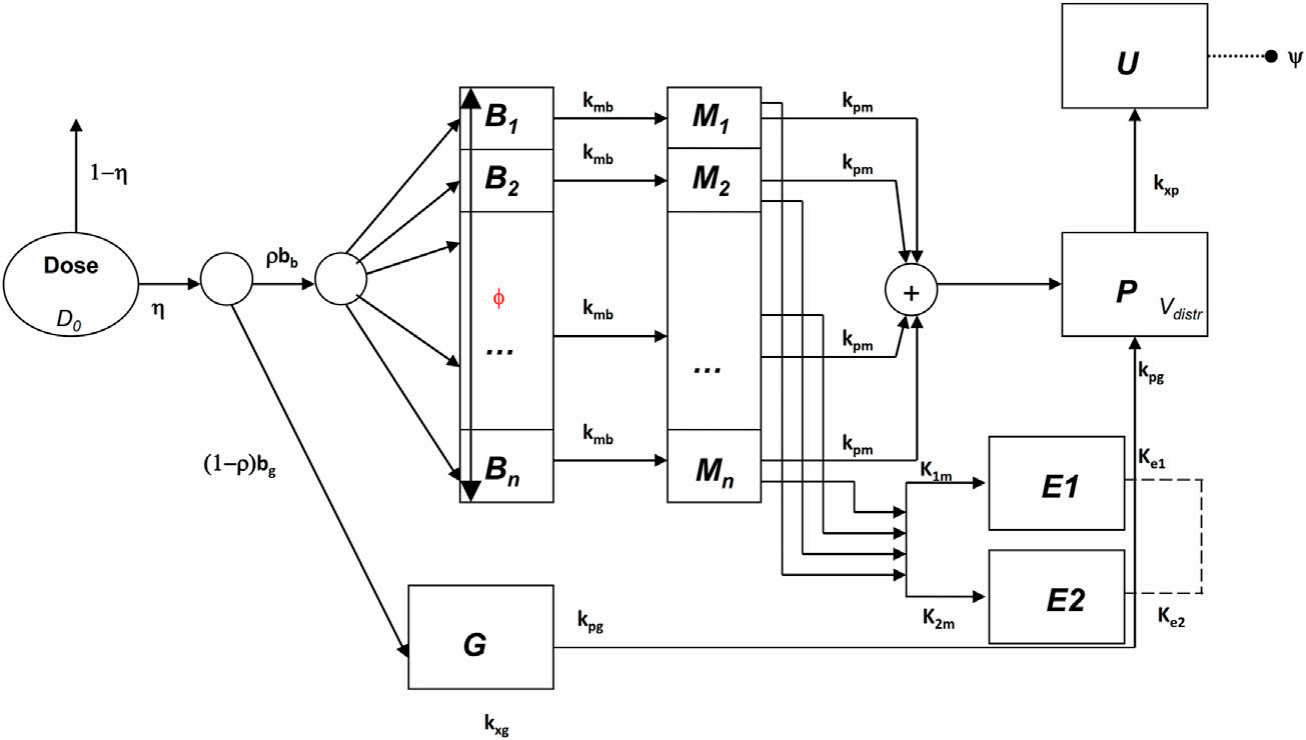
Schematic representation of the pharmacokinetic/pharmacodynamic model.

The drug dynamic effect over time is associated with the activity of the compound at a distal site E (which could represent the turnover rate of calcium ions in the sarcoplasmic reticulum), affected possibly by concurrent, parallel slow and fast delay mechanisms *E*_*1*_ and *E*_*2*_. See Figure 1 for a graphical representation.

The function *h(z)* in Eq. 7, as well as in Eq. 8, represents the β_2_-adrenergic receptor density, which, in the present formulation, is hypothesized to vary along the bronchi in an approximately linear fashion:

$$h\left(z\right)=h_{\min}+\alpha_{h}z$$ (12)

where *h*_min_ is the value of the receptor density at *z* = 0, and *α*_h_ is the approximately linear increase in receptor density per cm. The simpler (linear) formulation (Eq. 12) of the increasing receptor density down the bronchial depth resulted in any case numerically very similar to the original (Gaz et al., 2012) nonlinear formulation (Hill function). Again, the first term on the right-hand side of Eq. 8 represents the natural tendency of the disease condition to constrict bronchi towards the untreated state: its size depends on the effect entity as well as on the achieved level of broncho-dilatation. The second term on the right-hand side of Eq. 8 is 0 until the drug is administered [since *E*(*z*,0) = 0], then, as soon as the drug reaches the bronchial mucosa, the compound passes into the delay compartments and thence reaches the effect site.

All state variables and model parameters of the pharmacokinetic equations as well as of the pharmacodynamic equations are described in Table 3. Figure 1 reports the schematic diagram of the PK/PD model.

**TABLE 3:** Model pharmacokinetics and pharmacodynamics variables and parameters.

Pharmacokinetics variables/parameters			Numerical value first optimization/second optimization
Symbols	Units	Definition	
t	[hr]	Time in hours	[0–12]
z	[cm]	Distance of a point along the bronchial tree from the larynx	[0, 30]
D	[pmol]	Quantity of inhaled formoterol	29816.63 or 59633
f	#	Distribution density of the compound along z at time t = 0	—
B	[pmol]	(Aggregated) quantity of the drug in the bronchial compartment	—
M	[pmol]	(Aggregated) quantity of the drug in bronchial muscle fibers	—
G	[pmol]	Quantity of the drug in gastrointestinal tract	—
P	[pM]	Concentration of the drug in plasma	—
k_mb_	[hr^-1^]	Apparent first order transfer rate from bronchi to muscle	55
k_bm_	[hr^-1^]	Apparent first order transfer rate from muscle to bronchi	0
k_pm_	[hr^-1^]	Apparent first order transfer rate from muscle to plasma	82.5
k_mp_	[hr^-1^]	Apparent first order transfer rate from plasma to muscle	0
k_pg_	[hr^-1^]	Apparent first order transfer rate from gastrointestinal tract to plasma	1
k_xp_	[hr^-1^]	Apparent first order transfer rate from plasma to urine	1
k_xg_	[hr^-1^]	Apparent first order elimination rate from gastrointestinal tract	0
τ	[hr]	Delay between dose administration time and formoterol appearance in circulating system *via* gastrointestinal tract	0
V_distr_	[L/Kg]	Apparent distribution volume of formoterol	4
W	[Kg]	Body weight	—
η	#	Fraction of dose effectively inhaled, i.e that fraction not remaining in the spacer	0.9
ρ	#	Fraction of dose distributed to the bronchial compartment	0.30
b_b_	#	Bronchial bioavailability	0.99
b_g_	#	Gastric bioavailability	0.2
*φ*_*w*_	[cm^2^ hr^-1^]	Diffusion constant along bronchiolar wall	0.03
*E*_*1*_	[pmol]	(Aggregated) quantity of the drug in a delay muscle compartment	—
*E*_*2*_	[pmol]	(Aggregated) quantity of the drug in a delay muscle compartment	—
*E*	[pmol]	Quantity of the drug in a distal effect site	—
*k*_*mor*_	[hr^-1^]	Morbidity coefficient	0.5/to estimate
*k*_*med*_	[hr^-1^]	Drug efficacy coefficient	5
*h*_*min*_	#	Number of receptors in z = 0	0
*h*_*max*_	#	Maximum number of receptors	1500
*α*_*h*_	[cm^-1^]	Increment of receptors per cm	50
*k*_*1m*_	[hr^-1^]	Apparent first order transfer rate from muscle to the delay compartment E1	To estimate/0
*k*_*2m*_	[hr^-1^]	Apparent first order transfer rate from muscle to the delay compartment E2	To estimate/27.8
*k*_*e1*_	[hr^-1^]	Apparent first order transfer rate from compartment E1 to distal effect site E	To estimate/0
*k*_*e2*_	[hr^-1^]	Apparent first order transfer rate from compartment E2 to distal effect site E	To estimate
*k*_*xe*_	[hr^-1^]	Apparent first order elimination rate from compartment E	To estimate/12.7
*λ*_*1*_	[pmol^-1^]	Coefficient related to the return to morbidity conditions as depending on the quantity of the compound in the effect site	To estimate/0
*λ*_*2*_	[pmol^-1^]	Coefficient related to the effect of the drug in increasing the bronchial diameter	To estimate
*A*	#	The maximal restriction (as a fraction of 1)	To estimate
*b*	[cm]	Position of the maximal restriction along z	20
*c*	[cm]	Standard deviation of the gaussian curve which determines the width of the constriction	5

### The parameter estimation step

Due to evident *a-priori* unidentifiability, several parameters were kept fixed throughout the optimization process. Fixed model parameters were set to the original values used in Gaz et al. (Gaz et al., 2012) and are reported in Table 3. The free parameters to be estimated are related only to the pharmacodynamic part of the model and were fitted to the four sets (formoterol 12 μg at week 0, F12W0, and at week 12, F12W12; formoterol 24 μg at week 0, F24W0, and at week 12, F24W12) of experimental post-dose FEV_1_ observations over time derived during the visit after the inhalation of a single dose of formoterol and obtained by Figures 1, 2 of Pleskow et al. (2003) where the mean FEV_1_ values on the first (week 0) and last day (week 12) of the two treatment regimens (F12W0, F12W12, F24W0 and F24W12) are shown. Reliable values of the coordinates of the points were retrieved by using the software Plot Digitizer (https://plotdigitizer.com/app).

Initially, the parameter estimation process involved 8 of the pharmacodynamics parameters, whose estimates are reported in Table 4 (*A*, *k*_*1m*_, *k*_*2m*_, *k*_*e1*_, *k*_*e2*_, *k*_*xe*_, *λ*_*1*_, *λ*_*2*_). A Nelder-Mead simplex direct search was used for all optimizations and a weighted least squares estimation approach was followed. The precision of the estimates was computed by the asymptotic approximation:

$$\Sigma_{\hat{\theta }}=\sigma^{2}{\left(J^{T}WJ\right)}^{-1}$$

where

$$\sigma^{2}=\frac{1}{n-p}{\left(y-\hat{y}\;\right)}^{T}W\left(y-\hat{y}\right)$$

*n* and *p* are the total number of observations and the number of the free parameters, respectively, *J* is the Jacobian matrix with element (*i,j*) equal to $\frac{\partial y_{i,j}\left(\theta \right)}{\partial \theta }$ and where W is the diagonal matrix of weights whose elements are the inverse of the squared of the expectation.

**TABLE 4:** Estimates of the Model free parameters at the beginning (week 0) and at the end of the study (week 12) for the two treatment regimens, from the first optimization process.

Parameter	*A* [%]	*k*_*1m*_ [hr^−1^]	*k*_*2m*_ [hr^−1^]	*k*_*e1*_ [hr^−1^]	*k*_*e2*_ [hr^−1^]	*k*_*xe*_ [hr^−1^]	*λ*_*1*_ [pmol^−1^]	*λ*_*2*_ [pmol^−1^]
*F12W0*	38.8	1.16	49.4	14.1	0.15	13.5	7.9E-17	1.35
*F12W12*	35.9	0.63	9.3	17.1	0.46	16.2	1.9E-20	3.01
*F24W0*	40.8	2.55	64.6	15.3	0.12	16.8	7.2E-16	0.90
*F24W12*	38.5	1.69	9.58	45.8	0.33	4.12	1.1E-25	0.43

Since not all parameters could be estimated with precision (invertibility problems of the variance and covariance matrix at the optimum) the model was simplified by eliminating the slow delay mechanism E1. With the elimination of compartment E1, parameters *λ*_*1*_, *k*_*1m*_, *k*_*e1*_ also were discarded. The parameter *k*_*mor*_, representing the degree of morbidity, was allowed to vary and parameters *k*_*2m*_ and *k*_*xe*_ were set to the average values obtained in the previous fittings. In total, this second step required therefore the estimation of only four parameters: *A*, *k*_*mor*_, *k*_*e2*_ and *λ*_*2*_.

All computations were performed with the R2011b version of Matlab.

### FEV_1_ trend over time for the simulated populations

For each subject in the four simulated populations, the FEV_1_ trend over time (from time 0–12 h) was simulated according to the PK/PD model described above.

The parameter *A* in Eq. 6, representing the maximal restriction, is one of the model parameters to be estimated from Pleskow’s observations. For each simulated subject, however, it can be computed in percentage terms directly from the computed percentage of predicted FEV_1_ value (*PercFEV*_*1*_(*s*), where *s* indicates the simulated subject), made available from the *Simulation step* as described above.

The reasoning and the assumptions are as follows: let a bronchial section in normal conditions be approximated by a circumference with diameter equal to 1. Under a restriction (expressed in terms of percentage) of width *RestrPerc* the useful surface becomes:

$$S_{restr}=k\pi {\left(\frac{1-p\times A}{2}\right)}^{2}$$ (13)

where *p* is a parameter that translates the functional restriction in geometrical bronchial diameter reduction, and *k* is a constant value representing the total number of available surfaces along the bronchial tree (Gaz et al., 2012).

Since FEV_1_ can be computed directly as the product between the pressure delta and the useful surface, for a normal subject (under healthy conditions) the approximated expected FEV_1_ is:

$$FEV1_{norm}=\Delta P\times k\pi {\left(\frac{1}{2}\right)}^{2}$$ (14)

where ΔP is the pressure delta; the percentage of predicted FEV_1_ (given by the percent ratio between the actual FEV_1_, *FEV1*_*restr*_ and the expected FEV_1_, *FEV1*_*norm*_) for the subject *s* is therefore:

$$\begin{array}{l} PercFEV_{1}\left(s\right)=\frac{FEV1_{restr}}{FEV1_{norm}}\times 100=\frac{\Delta P\times k\pi {\left(\frac{1-p\times A\left(s\right)}{2}\right)}^{2}}{\Delta P\times k\pi {\left(\frac{1}{2}\right)}^{2}}\times 100\\ ={\left(1-p\times A\left(s\right)\right)}^{2}\times 100 \end{array}$$ (15)

from where the dependency on Δ*P* and *k* vanished, and from which it follows that:

$$A\left(s\right)=\frac{\left(1-\sqrt{PercFEV_{1}\left(s\right)/100}\right)}{p}$$ (16)

The unknown parameter *p* can be determined minimizing the following loss function:

$$\hat{p}=\min_{p}\left\{{\left[\bar{A_{F12W0}}\left(p\right)-{\hat{A}}_{F12W0}\right]}^{2}+\right.\left.{\left[\bar{A_{F24W0}}\left(p\right)-{\hat{A}}_{F24W0}\right]}^{2}\right\}$$ (17)

where $\bar{A_{F12W0}}$ and $\bar{A_{F24W0}}$ are the averages of *A(s)* computed for the 10,000 simulated subjects in the F12W0 population and for the 10,000 simulated subjects in the F24W0 population respectively according to Eq. 16, and where ${\hat{A}}_{F12W0}$ and ${\hat{A}}_{F24W0}$ are the estimates of parameter *A* in Eq. 6 obtained by fitting the model onto the datasets F12W0 and F24W0 respectively with the first optimization step.

The function *A(s)* for the two populations at week 12 (populations F12W12 and F24W12) can be computed hypothesizing that the percentage of predicted FEV_1_ for the two simulated populations is different at week 12 with respect to week 0 due to an additive factor Δ (with Δ ≥ 0) expressing the effect of treatment during the course of the study period. The parameter Δ is determined, with *p* fixed at the estimated value $\hat{p}$ , by minimizing the following expression:

$$\begin{array}{l} \hat{\Delta }=\min_{\Delta }\left\{{\left[\bar{A_{F12W12}}\left(PercFE{V_{1}}_{,F12W12}\left(\Delta \right),\hat{p}\right)-{\hat{A}}_{F12W12}\right]}^{2}\right.\\ \left.+{\left[\bar{A_{F24W12}}\left(PercFE{V_{1,}}_{F24W12}\left(\Delta \right),\hat{p}\right)-{\hat{A}}_{F24W12}\right]}^{2}\right\}. \end{array}$$ (18)

Once having obtained for each simulated subject his/her own percentage restriction, other model parameters were set to the specific value for the subject when available (age, gender, height, expected FEV_1_). The remaining parameters were set to the estimated values from the *Parameter Estimation step* or were kept fixed to their original values as reported in Table 1. Eqs 17, 18 proved necessary in order to estimate the Pleskow’s populations features in terms of percentage of restriction. In an actual clinical setting, the patients might be undergone a sequence of spirometry tests after drug administration, and the model can be fitted to the patient’s observed data for estimating the parameter *A*, which indeed represents the degree of bronchial restriction.

## Results

**FIGURE 2:**
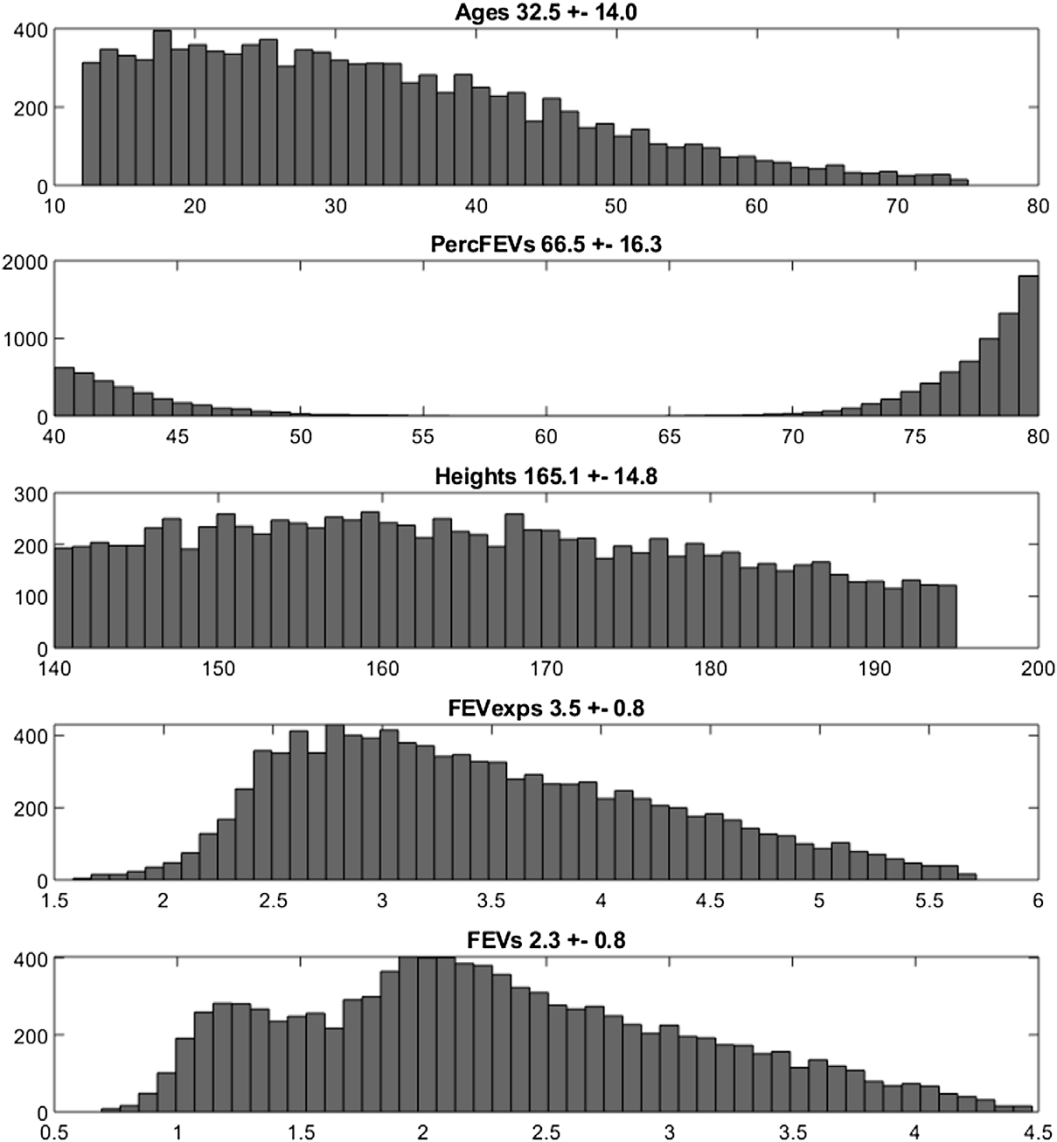
Histograms of frequencies of the simulated demographic and related-disease characteristics for the population undergoing 12 μg of formoterol *via* Aerolizer.

**FIGURE 3:**
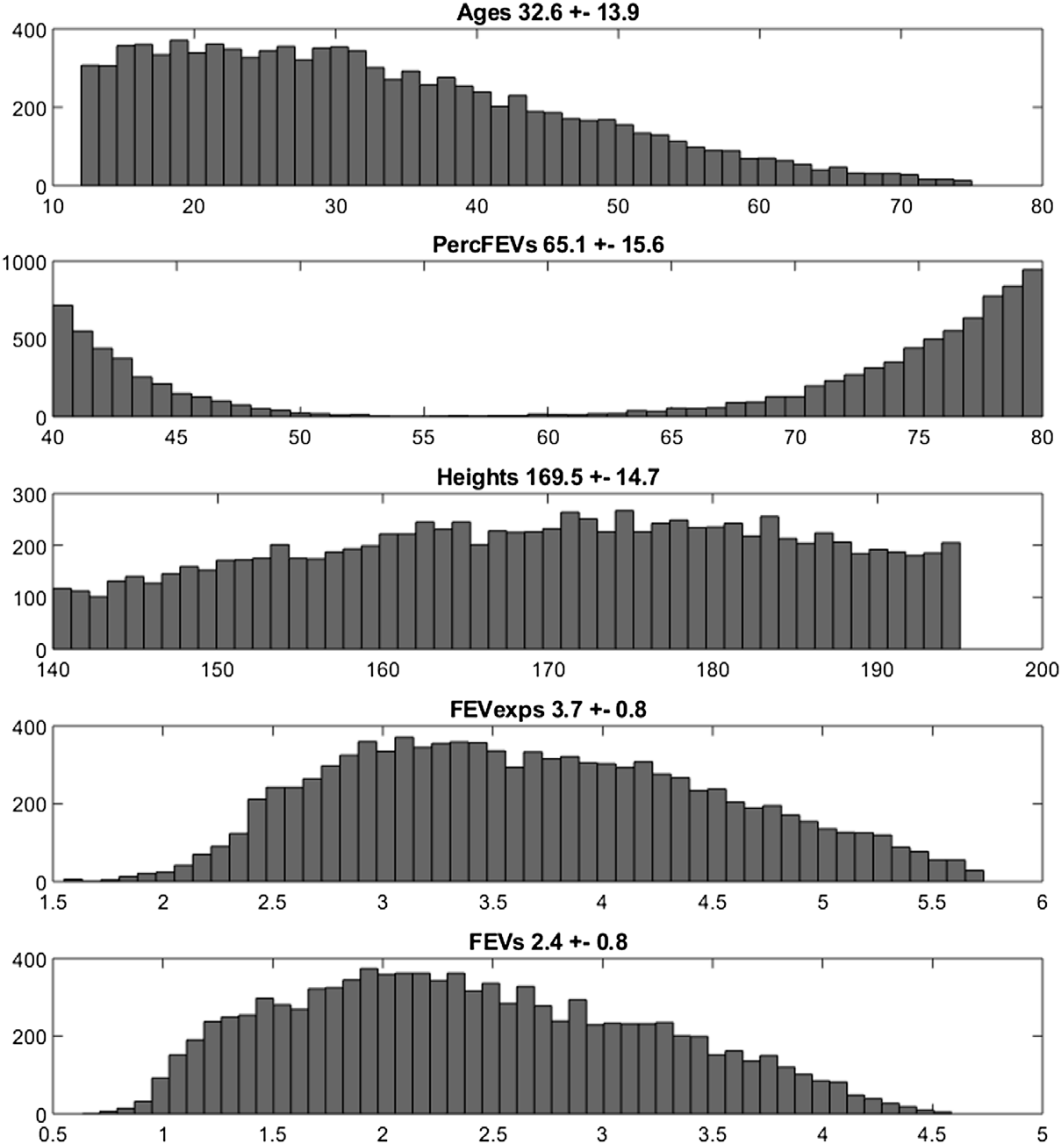
Histograms of frequencies of the simulated demographic and related-disease characteristics for the population undergoing 24 µg of formoterol *via* Aerolizer.

The empirical distributions of the demographic and disease-related characteristic (age, height, expected FEV_1_, observed FEV_1_ and percentage of predicted FEV_1_) of the two simulated populations are shown in Figures 2, 3 (formoterol 12 and 24 μg, respectively). Average heights and the relative standard deviations were estimated to be 165.1 ± 14.8 and 169.5 ± 14.7 cm for 12 and 24 μg formoterol treatment. The values in the two gender subsamples resulted to be 169.2 ± 15 cm and 161.1 ± 13.5 cm for males and females, respectively, in the 12 μg formoterol population. Values for males and females in the 24 μg formoterol population were 172.5 ± 14.5 and 165.7 ± 14.1 cm, respectively. The obtained values approximate the average gender-specific height in North and Central America, where the study is supposed to have been conducted: 173 cm and 160 cm for males and females, respectively (https://www.worlddata.info/average-bodyheight.php). Note that there is a difference between the average heights obtained in the 12 μg and 24 μg formoterol populations: the mean heights are lower in the lower-dosed population. Since population heights were not reported in Pleskov’s work, it is not possible to say whether the simulated populations closely resemble the original populations in terms of heights; however, the difference could be due to the fact that a lower dose was mainly given to younger individuals.

**FIGURE 4:**
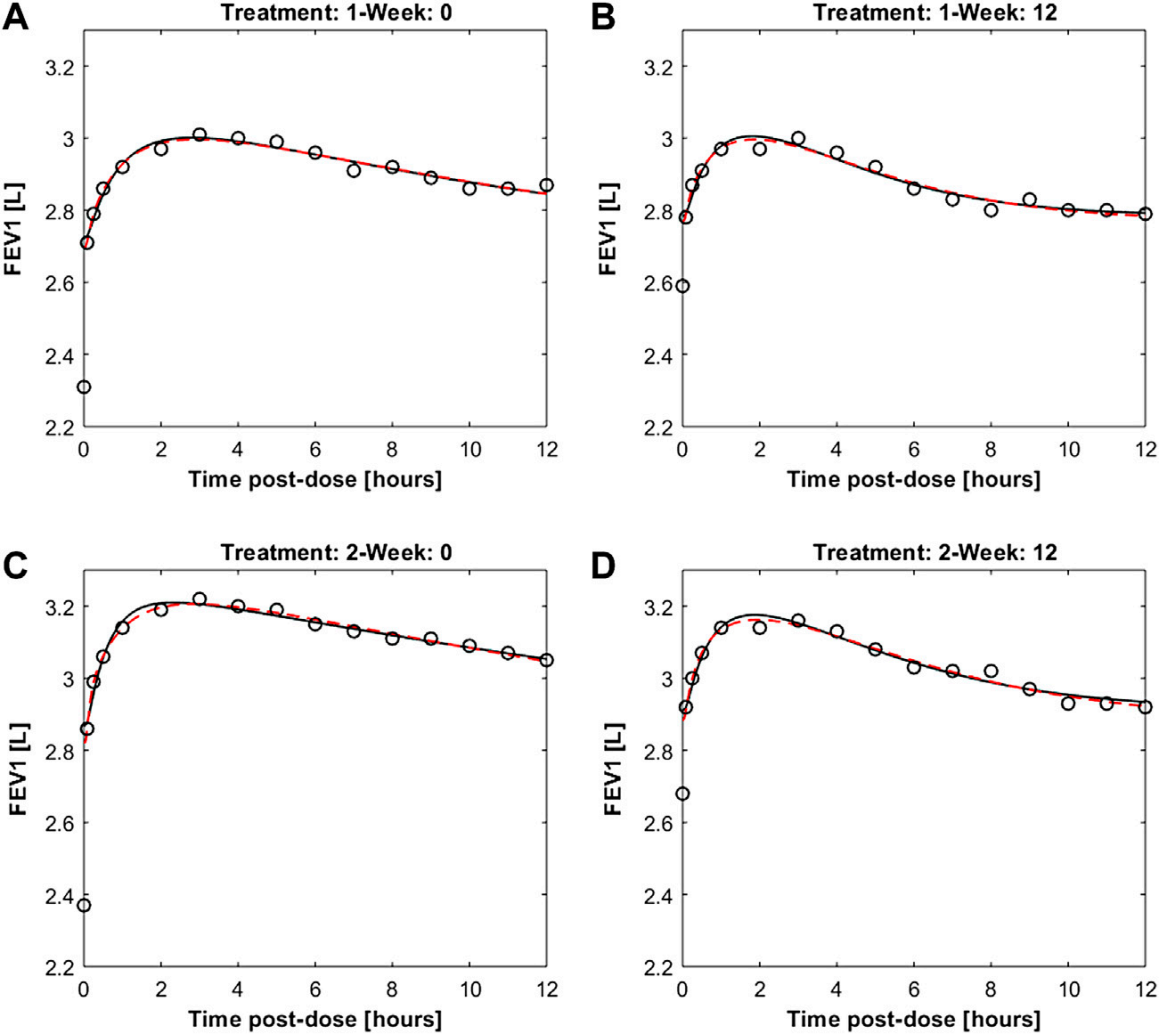
Panels **(A,B)** report observed and predicted mean FEV_1_ on the first day and at week 12 of double-blind treatment with formoterol 12 μg delivered *via* Aerolizer respectively; panels **(C,D)** report instead observed and predicted mean FEV_1_ on first day and at week 12 of double-blind treatment with formoterol 24 μg respectively.

Figure 4 reports the observed and predicted post-dose values of FEV_1_ over time in the two treatment regimens at week 0 and at the end of the study period for both the two fitting procedures. The dashed red lines are the predictions obtained estimating the eight pharmacodynamics parameters; continuous black lines represent the predictions obtained with only four free parameters. Panels A and B report the expected responses for treatment formoterol 12 μg at week 0 and at week 12 respectively; panels C and D report results related to treatment group formoterol 24 μg. The estimates of the model free parameters obtained in the two optimization procedures are reported in Tables 4, 5.

**TABLE 5:** Estimates of the Model free parameters along with the respective standard deviations and coefficients of variation, at the beginning (week 0) and at the end (week 12) of the study for the two treatment regimens, from the second optimization process.

Parameter	*A* [%]	SD	CV [%]	*k*_*mor*_ [hr^−1^]	SD	CV [%]	*k*_*e2*_ [hr^−1^]	SD	CV [%]	*λ*_*2*_ [pmol^−1^]	SD	CV [%]
*F12W0*	37.8	0.41	1.09	0.55	0.11	20.40	0.16	0.03	16.16	2.19	0.60	27.32
*F12W12*	35.3	0.37	1.04	0.51	1.12	221.03	0.58	1.20	208.45	0.76	1.69	222.36
*F24W0*	39.1	0.37	0.95	0.87	0.12	13.54	0.12	0.01	10.62	2.47	0.48	19.48
*F24W12*	37.5	0.44	1.17	0.69	0.22	31.79	0.36	0.09	24.73	0.63	0.25	39.52

SD, standard deviation; CV, coefficient of variation.

From Table 4 the four estimates of restriction were used to estimate once the parameters *p* and *Δ* from Eqs. 17, 18, useful for the computation of the percentage of restriction for each individual of the simulated populations. Table 5 reports the final estimates of the free model parameters when only four parameters were allowed to vary. The estimates of the restrictions continue to be coherent with the mean values obtained from the 10,000 simulated subjects of the two populations as described in the subsection “*FEV*_*1*_ *trend over time of the simulated populations*” above. For formoterol 12 μg the estimates were 37.8% and 35.3% at the beginning and at the end of the study period, respectively versus the average values over the 10,000 subjects of 39.0% at week 0 and 36.4% at week 12. For formoterol 24 μg the estimated values were 39.1% and 37.5% whereas the computed averages were 40.6% and 38.0%.

All free parameters were identifiable in all experimental situations, with the exception of the 12 μg experimental regime at week 12, where the Coefficient of Variations (CVs) resulted to be larger than 200% for all the free parameters except for parameter *A* (percentage of restriction). Conversely, in the F12W0, F24W0 and F24W12 experimental situations CVs varied from a minimum of 0.96% to a maximum of 39.5%.

**FIGURE 5:**
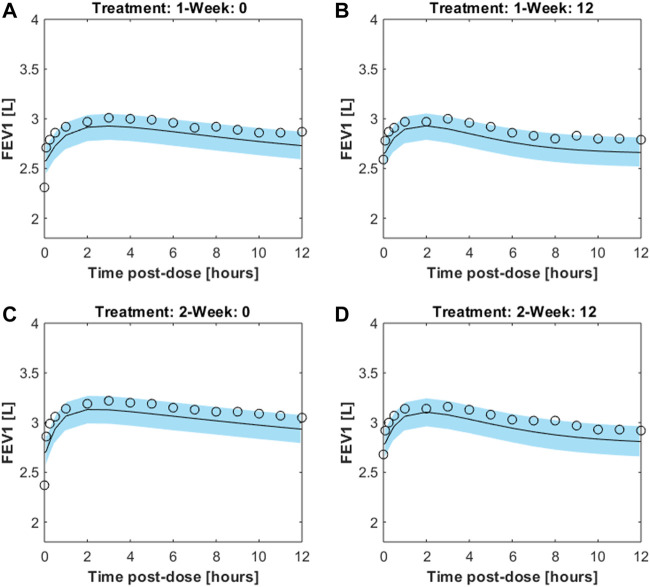
Results from 200sets of 100simulated individuals, each. For each simulated individual, trend over time of FEV_1_ was simulated by running the model with parameters set to the fixed and estimated values from the second fitting procedure, except for the specific individual parameters age, height, gender and percentage of restriction. Continuous black lines are the mean trends over time from the 200sets whereas light-blue areas represent the 95% confidence bands of means (between its 2.5% and 97.5% percentiles). Results are showed for the two experimental regimes both at week 0 and at week 12 for double-blind treatment with formoterol 12g [panels **(A,B)**, respectively] and with formoterol 24g [panels **(C,D)**, respectively].The image used in Figures 5 and 6 have part labels AD; however, the description is missing in the caption. Could you clarify this? Provide revised files if necessary.

The trend over time of FEV_1_ after drug administration was simulated for 200 sets of 100 individuals each. The individual trend was obtained by running the model with parameters set to the fixed and estimated values from the second optimization procedure, except for some specific individual parameters (age, height and gender, useful for the computation of the expected FEV_1_; percentage of restriction). For each set the average trend was computed along with its 2.5% and 97.5% percentiles. Figure 5 shows the mean FEV_1_ trends over time (black lines) from the 200 sets, as well as the 95% confidence bands of means for the simulated populations for the two experimental regimes both at week 0 and at week 12; Pleskow’s observations (circles) are also reported.

**FIGURE 6:**
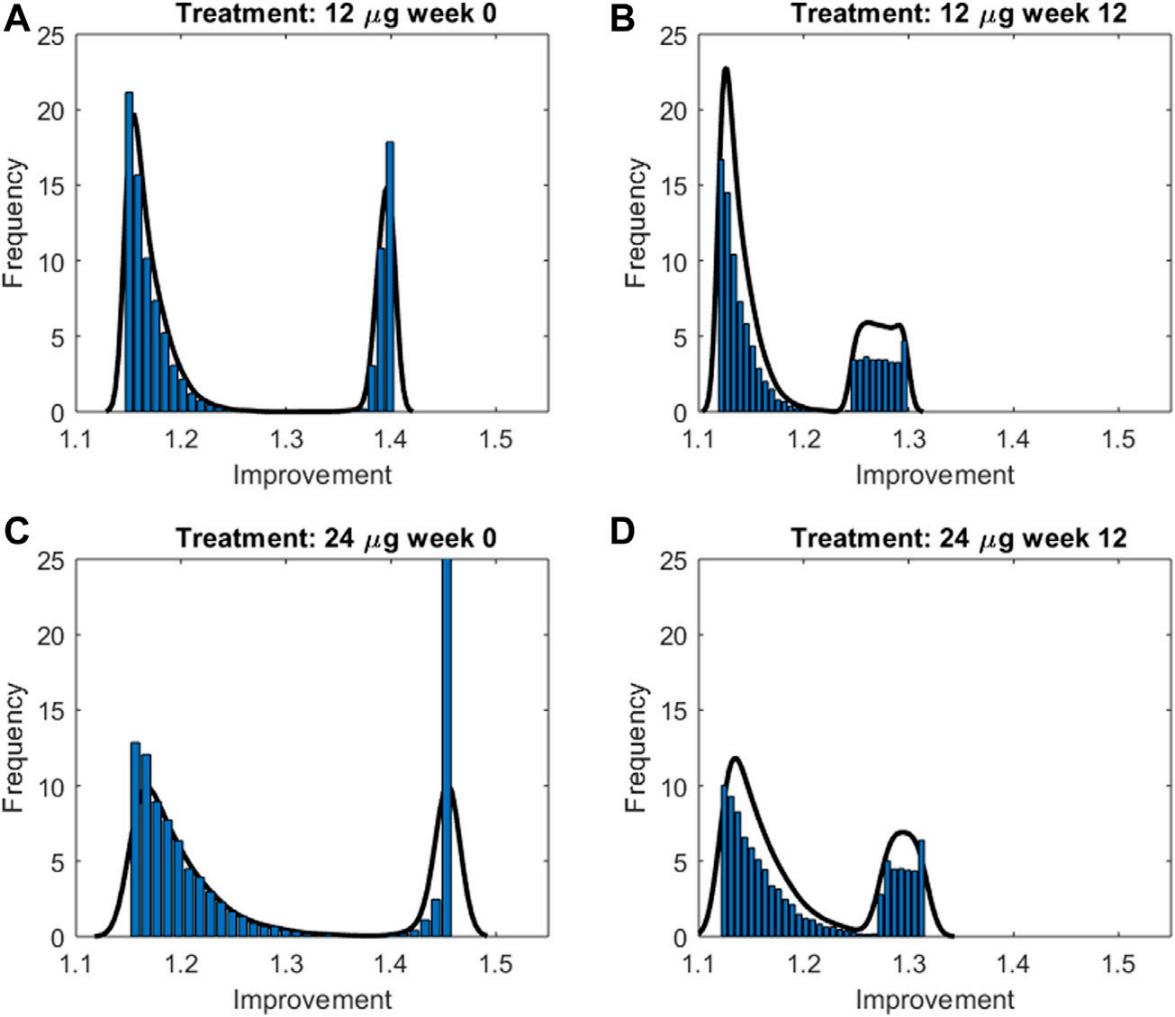
Frequency distributions along with the relative kernel density estimation of the improvement indices computed for the two treatment groups at week 0 and 12 for double-blind treatment with formoterol 12g [panels **(A,B)**, respectively] and with formoterol 24;g [panels **(C,D)**, respectively].

In order to summarize the efficacy of treatment, for each simulated subject an “Index of Improvement” was computed as the ratio between the average predicted FEV_1_ over time and the basal FEV_1_ level. Figure 6 reports the frequency distributions of the index along with the relative kernel density estimation of the distributions. All the distributions show a bimodal shape, in all likelihood reflecting the hypothesized bimodal distribution of asthma severity. As an example, let consider the 24 μg formoterol population at week 0 (panel A) and divide the population into two subpopulations: individuals who show a large improvement (larger than 1.35) and individuals with a small improvement (smaller than 1.3). The analysis of the characteristics of the individuals belonging to the two different distributions showed that the two subpopulations do not differ significantly with respect to distribution of gender, height, age and hence expected FEV_1_ (males: 55.9% vs. 55.8%, height: 169.5 cm vs. 169.6 cm; age: 32.9 years vs. 32.4 years; expected FEV_1_: 3.7 L vs. 3.7 L in the group with large and small improvement, respectively) whereas the two subgroups show a very different baseline FEV_1_ expressed as percentage of predicted FEV_1_: 44.9 ± 2.5% vs. 77.5 ± 3.9% in the subpopulation with large improvement and in the subpopulation with small improvement, respectively, which translates into a larger percentage maximal restriction: 67.2 ± 3.7% vs. 24.4 ± 4.6%, respectively.

**FIGURE 7:**
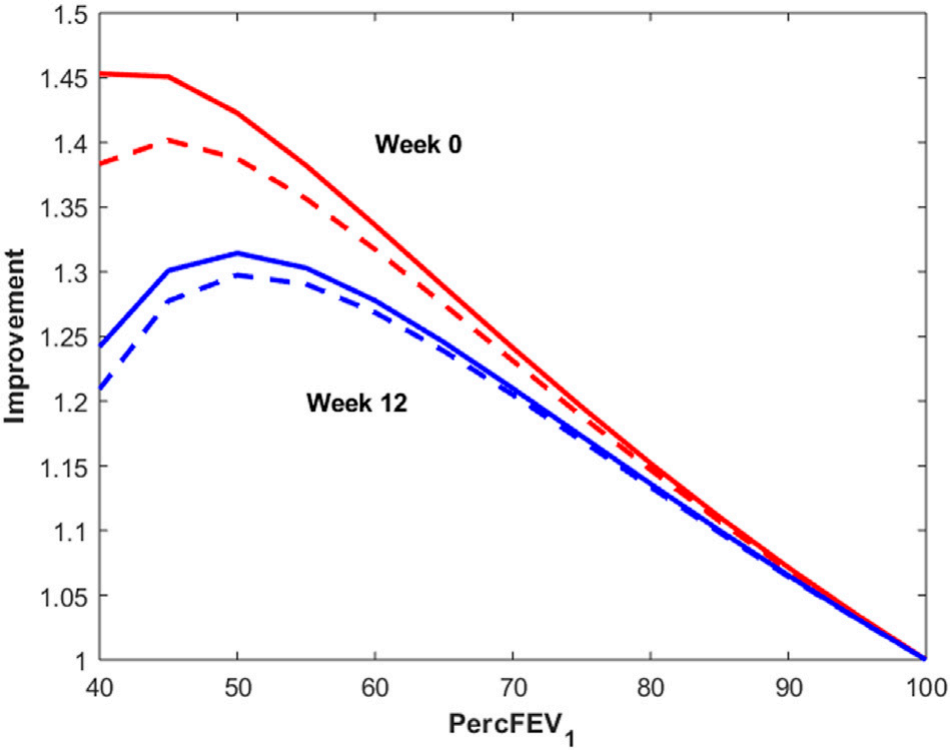
Improvement index as a function of percentage of predicted FEV_1_ for a male subject, 175 cm height, aged 30 years, undergoing 12 μg (dotted lines) or 24 μg (continuous lines) of inhaled formoterol at week 0 (red lines) and week 12 (blue lines).

Figure 7 reports instead the Improvement index as a function of percentage of predicted FEV_1_ for a male subject, 175 cm height, aged 30 years, undergoing 12 μg (dotted lines) or 24 μg (continuous lines) of inhaled formoterol at week 0 (red lines) and week 12 (blue lines). As expected, the Improvement decreases with increasing percentage of predicted FEV_1_, highlighting a larger effect of treatment both in the presence of worse conditions and at the beginning of the experimental period.

## Discussion

Simulation of the PK/PD behavior of populations of interest, based upon mechanistic, predictive mathematical models, can be a very useful tool for inferring the efficacy of novel therapeutic schemes, in particular when designing clinical studies for drug candidate testing. Drug dosages, treatment schedules, sample characteristics and study arms can be planned with a somewhat higher degree of confidence in the eventual success of the investigation when preliminary simulations support the choices made and give approximate indications about the results that can be obtained. The predictions obtainable from mathematical models may have very different properties when extrapolating available information to novel investigational areas, be it due to varying populations, varying physico-chemical characteristics of the compound or varying routes and schedules of administration. In this case, the more physiologically structured the model is, the more likely it is that its predictions will be accurate upon extrapolation.

When faced with the complexity of modeling airway distribution and deposition of inhaled droplets, direct approaches like Computational Fluid Dynamics simulation are appealing for the precision and detail with which they are able to reconstruct actual gas flow in topographically accurate segments of the bronchial system. This approach, essentially due to the heavy inherent computational load, is best suited for the detailed reconstruction of flow in small subsegments (a few branching generations) of a specific subject’s bronchial tree. On the other hand, if information on population efficacy and its variability is sought, a simpler approach is needed: a simplified bronchial geometry model could retain sufficient physiological plausibility to afford quantitative extrapolation while remaining sufficiently compact to allow meaningful adaptation to records of FEV_1_ tests from actual subjects participating in a clinical trial.

In the present work, the pharmacodynamics part of a previous overall PK/PD model (Gaz et al., 2012) was improved and its new formulation was validated on previously published data obtained from a sample of adolescent and adult asthmatics (Pleskow et al., 2003).

The pharmacokinetics part of the present model includes bronchial mucosa and bronchial muscle, represented not as homogeneously stirred compartments, but as distributed systems endowed with local deposition and transport as well as with longitudinal diffusion.

The pharmacodynamics part of the model describes the dynamic effect of local quantities of the compound to relax bronchial smooth muscle and increase local bronchial diameter over time. The diameter variation leads in turn to a corresponding variation of local bronchial resistance, and hence, upon integration throughout bronchial tree depth, to a representative airflow, inversely related to overall resistance through Ohm’s law. The previously presented model (Gaz et al., 2012) needed to be modified in order to be able to adapt acceptably to measured average FEV_1_ profiles: initially, two parallel delay compartments, one faster and one slower, were introduced between smooth muscle and effect site. This modification appeared to be essential to reproduce the four sets of experimentally observed FEV_1_ values as reported in Pleskow et al. (2003), recorded on two different patient samples, undergoing respectively 12 μg and 24 μg of formoterol dry powder administered twice daily, and taken at week 0 and after 12 weeks of a double-blind treatment period. The observed FEV_1_ profiles exhibited a fast initial rise and a subsequent progressive slow decay of the treatment effect on FEV_1_, which could not be reproduced by the model in its original version (Gaz et al., 2012). The introduction of the two compartments gave rise to a very good adaptation of the model (allowing 8 free parameters to vary) to the observed averages, as shown in Figure 4, suggesting that the pharmacologic effect is mediated by intra-cellular mechanisms acting over time at different speeds. Since not all parameters could be estimated with precision the model was simplified by eliminating the slow delay mechanism E1. With the elimination of compartment E1, some parameters also were discarded, while the additional parameter *k*_*mor*_, representing the degree of morbidity, was estimated too for a total of four free parameters. Results from this second optimization phase showed that the model was a-posteriori identifiable. All free parameters were indeed identifiable in all experimental situations, with the exception of the 12 μg experimental regime at week 12. This might be an effect of acquired tolerance to the drug when administered at low doses. The available dataset may not be sufficient to identify model parameters in situations where the drug does not produce a marked effect. In the case of the administration of 24 μg, on the other hand, despite a treatment period of 12 weeks, the drug, given at higher doses, continues to produce an effect that the model is able to accurately represent.

However, the estimates are in line with what expected. In all cases, in fact, after 12 weeks of treatment both the percentage of restriction (*A*) and the degree of morbidity (*k*_*mor*_) are reduced, highlighting an improvement in the patient’s condition; parameter *λ*_*2*_ also decreased from week zero to week 12: smaller values of the parameter produce a lesser effect of the drug, presumably indicating a progressive tolerability to the drug.

*A priori* identifiability would have allowed to determine if the parameters could be in theory uniquely estimated. While it would have been a very substantial addition to model development, identifiability analysis for complex nonlinear models is a difficult mathematical task. DAISY (Saccomani et al., 2019) is a software tool for testing the identifiability of biological models, but, to our knowledge, it was developed to treat ODE models and it would have been difficult to adapt it to the present problem which deals with a mixed compartmental and distributed PK/PD model (see for example Eqs 3, 4). In this particular case or in the case of even more complex models, it is in any case possible to carry out an a-posteriori model identifiability study, with the computation of the (asymptotic) standard deviations of the parameter estimates, using approximate solutions for the derivation of the variance and covariance matrix of the parameter estimates obtained.

The introduction of an explicit drug tolerance term would have obviated the need of the separate estimation of some of the free parameters of the model for different experimental conditions. The consideration of a dose-dependent effect, in fact, would have made it possible to conduct a single, albeit more complex, parameter estimation procedure over the entire experimental dataset. Future work may address this modelling of a dose-dependent effect.

Once an appropriate model appeared to have been found, it was necessary to simulate a population of virtual patients similar to those actually observed by Pleskow et al. (2003), in order to obtain individual responses to the administration of the compound and subsequent sample average trajectories and confidence bands.

While reproduction of the demographic characteristics of the Pleskow’s population (age, distribution of gender, height and expected FEV_1_, derived as a function from the previous variables) was not difficult, finding the distribution of the percentage predicted FEV_1_, as reported by Pleskow, proved more of a challenge. It was in fact found that a bimodal distribution was necessary in order to reproduce the reported mean with its quite large standard deviation: monomodal distributions, or even a completely uniform distribution between the reported acceptance extremes (40%–100%), exhibited a much smaller variance than reported. The conclusion appeared therefore inescapable that the studied patient sample in Pleskow’s work (Pleskow et al., 2003) was composed of subjects with either very mild or with moderate-to-severe asthma, with little intermediate disease severity.

Results show a very good match of the considered baseline characteristics, between real and virtual subjects, as reported in Table 1. Once the virtual population was matched with the real sample of patients, the model was used to predict the FEV_1_ profile for each virtual subject, as depending from that subject’s randomly generated gender, size, and degree of baseline bronchoconstriction.

The validity of the model was assessed by means of visual inspection of the adaptation of the average FEV_1_ predictions (reported together with the corresponding 95% confidence bands) of virtual subjects to the corresponding observed averages reported by Pleskow et al. (Figure 5) (at week 0 and weeks 12 study periods, for both 12 and 24 μg dosages): all panels in the figure show a good fit of the predictions to the data (even if they slightly underestimate the observed trends), suggesting that the assumptions underlying the model reflect with good approximation the average physiological behaviour of the patient sample by Pleskow.

It must be underscored that the assumption that the mean of Pleskow’s patients’ time-courses is representative of the shape of the individual time-courses themselves may not necessarily be true. Further, even if single-subject time courses did exhibit a qualitatively similar behaviour to the mean time-courses reported in Pleskow et al. (2003), it is not necessarily true that the mean of a large sample of simulations (20,000 simulations whose variability reflects patient sample heterogeneity as reported in Pleskow’s demographics tables) would be superimposed, or even parallel, to the observed mean trend. That this is in fact the case supports the plausibility of the model as a physiologically coherent description of the experiments.

In order to better represent the effect of the compound on the response variable, an improvement index was computed for each simulated patient for each experiment, as the ratio between the average FEV_1_ over experiment time and the subject’s pre-dose FEV_1_ (before formoterol administration). The distribution of the index, summarized by its kernel density, revealed the presence of a sub-population of responders for which the improvement was larger. The identification of these stronger responders, in terms of their baseline characteristics, showed that this group was denoted by a more severe grade of morbidity: subjects in this group started with a much smaller percentage of predicted FEV_1_ than the rest, accompanied by a rather small variance in grade of morbidity.

It is interesting to observe that the modal peaks (Figure 6) are essentially superimposed at the low-improvement modes (index of improvement from about 1.12 to about 1.17), indicating that the many patients of the low-morbidity subpopulation respond very moderately to treatment, independently of study period (week 0 or week 12) and independently of dosage (12 or 24 μg). Conversely, the high-improvement modes show differences in improvement index position depending on dosage, treatment with 24 μg determining a larger improvement than treatment with 12 μg at each study period; high-improvement modes are actually higher for week 0 than for week 12, which could be consistent with a progressive therapeutic effect throughout the several weeks, determining better baseline and consequently smaller possibility of improvement at 12 weeks Figure 7 shows the relationship between baseline bronchial status and improvement index, for both 12 and 24 μg doses at week 0 and at week 12 for a given subject. From Figure 7 it clearly appears how the difference of effect between doses diminishes as the subject’s condition improves. The fact that the 24 μg dose appeared much better than the 12 μg dose in the more compromised subgroup, particularly at week 0, could have implications for the choice of subjects to whom higher initial dosages of formoterol may be immediately administered, possibly without titration through smaller dosages.

In conclusion, the proposed model appears to capture plausibly the essential mechanics of bronchial dilatation in response to inhaled bronchodilators, and reproduces well available pharmacodynamic observations. The model may therefore be used for making informed guesses during the planning phase of clinical trials and for comparing experimental observations from different treatments when the identification of the underlying physiological mechanisms is of interest.

## Data Availability

The original contributions presented in the study are included in the article, further inquiries can be directed to the corresponding author.
